# Supplementary material for: Antibody Properties Associate with Clinical Phenotype in LGI1 Encephalitis
Source: Cells. 2023 Jan 11;12(2):282. doi: 10.3390/cells12020282 (PMC9856817; doi:10.3390/cells12020282)
Supplement: Supplementary file 1 [file cells-12-00282-s001.zip › cells-2002446-supplementary.pdf]

Legend to supplementary material:

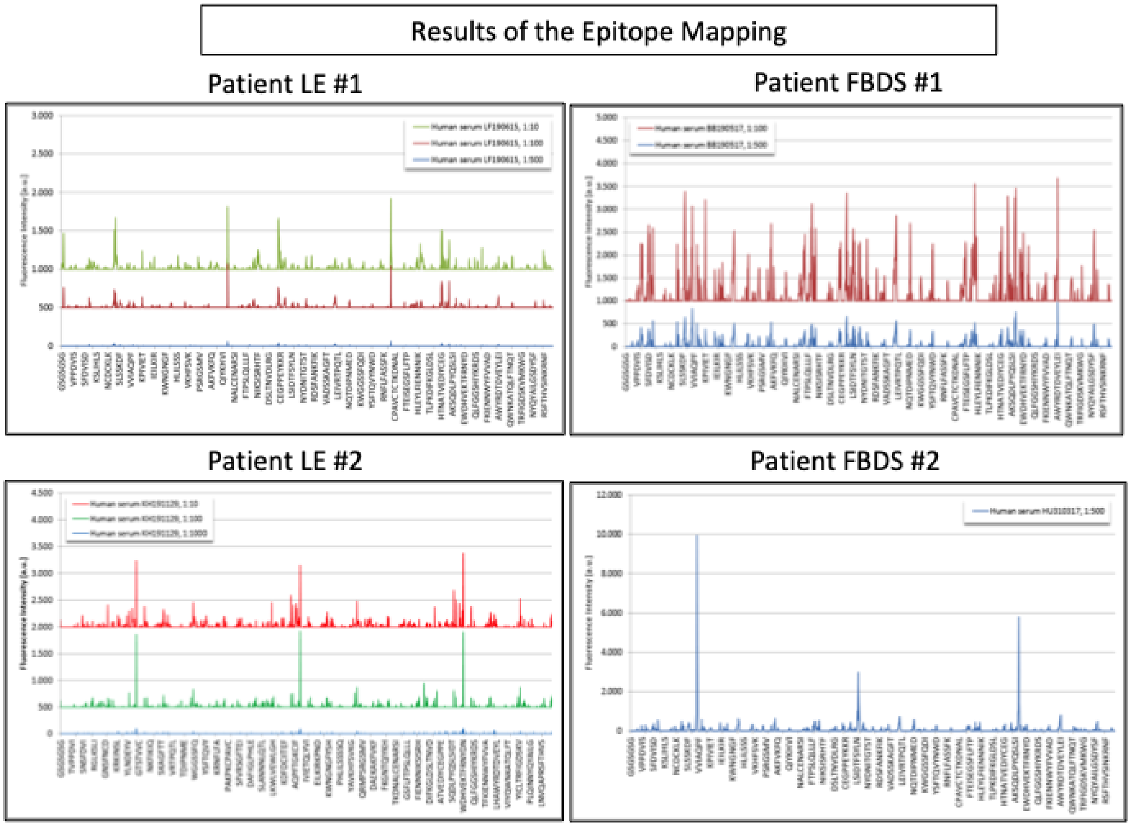

Figure S1: Result of the epitope mapping. Three conditions (7 aa, 10 aa, 13 aa) were used to identify the respective epitopes. Those regions, which showed high fluorescence intensity in all three conditions, were chosen as epitope (see method section).

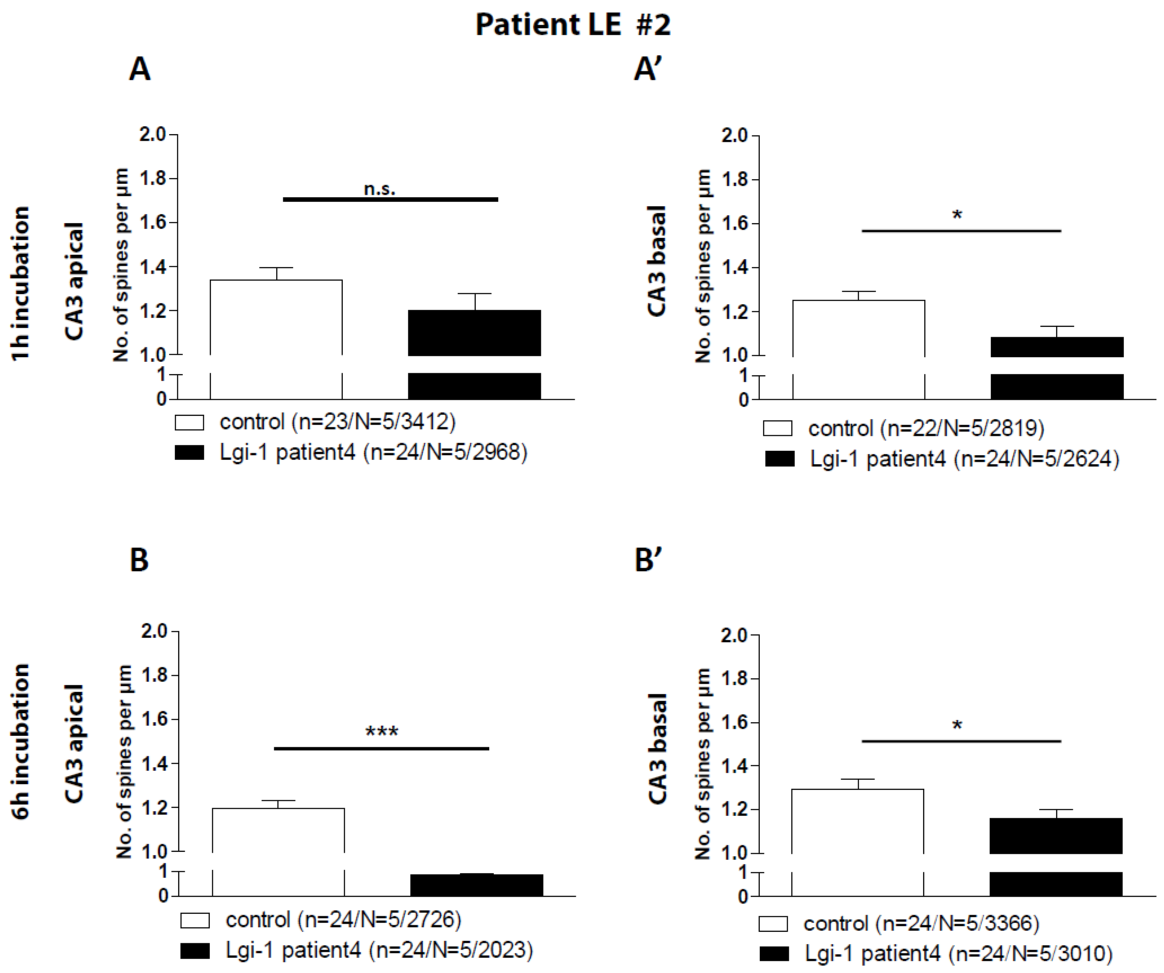

Figure S2: Quantified spine density after short and long ab application of patient LE#2 in CA3. Comparable spine density of CA3 pyramidal cells after 1 h at apical 2nd or 3rd order dendritic branches (A), but reduced spine density at the basal dendritic compartment (A'). 6 h treatment with IgGs of patient LE#2 reduced apical (B) and basal (B') and spine density Data presented as mean ± SEM, n = number of dendrites, N = number of animals, spines = number of total spines. Significant results are marked with an asterisk.

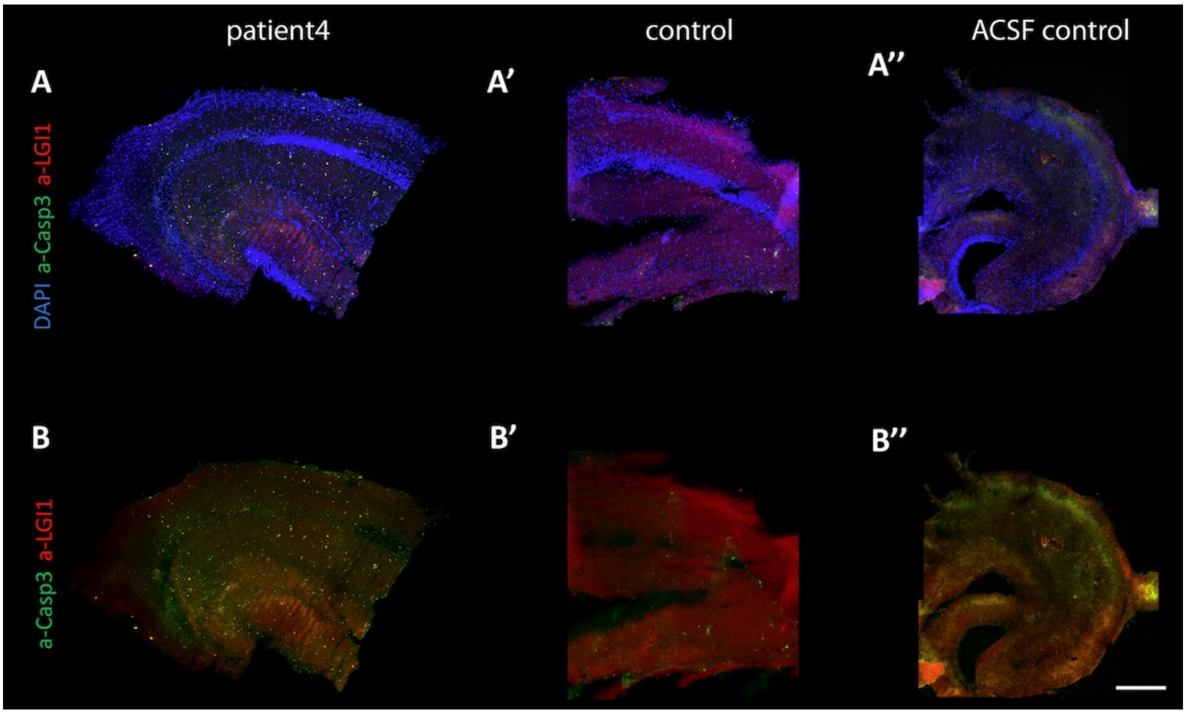

Figure S3: Representative images of anti-Caspase3 staining in hippocampal slices. Strong colocalziation of IgG fractions of patient LE#2 with Caspase3 (A, B), but not upon treatment with IgGs from healthy controls (A', B') or in ACSF (A'', B'') perfused slices. Scale bar = 200 µm.
